# Influence and compensation of connection characteristics on shaking table control performance

**DOI:** 10.1038/s41598-024-57239-z

**Published:** 2024-03-22

**Authors:** Chunhua Gao, Cun Li, Zihan Yuan, Yifei Sima, Mingyang Wang

**Affiliations:** College of Architecture and Civil Engineering, Xinyang Normal University, Xinyang, 464000 China

**Keywords:** Shaking table, Multi-parameter control, Connection characteristics, Compensation algorithm, Civil engineering, Mechanical engineering

## Abstract

When testing earthquake simulation shaking tables, it is commonly assumed that the test load, table, and actuator are integrated, which differs significantly from the actual situation and negatively impacts the accuracy of system waveform reproduction. This paper simplifies the connection between the three as a spring-damping model for simulation modeling. The effects of different load mass, different connection intrinsic frequency, different connection damping ratios, and other factors on the control performance of the system are analyzed, and based on the results of the analysis, a method to improve the effects of the connection characteristics on the performance of the system, called flexible connection reaction force compensation algorithm, is proposed. Resonance peaks caused by flexible connections reduce the effective bandwidth. To broaden the bandwidth and enhance system stability, the paper introduces a flexible connection force compensation algorithm based on a multi-parameter control algorithm to compensate for the interaction force caused by the connection characteristics. This compensation strategy expands the effective bandwidth, eliminates resonance peaks, improves the waveform correlation coefficient (CC), and reduces the root-mean-square error (RMSE).

## Introduction

An earthquake shaking table is one of the most widely used devices in the laboratory for earthquake simulation^1,2^. Traditional modeling approaches view the load table as an utterly rigid connection. However, in reality, the load-to-table connection is not entirely fixed, resulting in structural and non-structural loads being affected by the interaction between the shaking table and the load when applying the target excitation at the bottom of the shaking table^3,4^. Blonde et al.^5^, Dyke et al.^6^, and Conte et al.^7^ confirmed by experiment and simulation, respectively, that the effect of the load-table interaction force on the system is not negligible and that there is a robust dynamic interaction between the payload and the oil column of the actuator. The specimen characteristics affect the construction of the dynamic equations of the shaking table system. When nonlinear conditions such as tilting moments generated by eccentric loads occur, they narrow the system’s operating bandwidth^8^. Wang et al.^9^ developed an analytical transfer function matrix (TFM) for a shaking table and an eccentric load to analyze the interaction effects between the load and the table, investigated the impact of different mass ratios, moment of inertia ratios, and eccentricity distance ratios on the TFM, and proposed an evaluation of the effects to determine whether the impact on the system is negligible or not. In addition to the load characteristics, the system is affected by nonlinear factors such as the coupling of multi-axis and multi-dimensional actuators and multiple shaking tables^10^. In the area of multi-axis, multi-dimensional single shaking tables, Seki et al.^11^ proposed a modeling and control method for a two-dimensional shaking table system based on the geometrical arrangement and the equations of motion to introduce feed-forward compensation for a two-dimensional shaking table system model with multiple actuators and specimens to eliminate the moment effect. Wang et al.^12^ examined the interaction between a unidirectional, biaxial shaking table and a structure. They established the transfer function matrix (TFM) between the shaking table and the structure and proposed a real-time compensated control strategy. The strategy fully indemnified the effects of different mass ratios, damping ratios, frequencies, and moment of inertia ratios of the shaking table in the frequency domain. It also improved the accuracy of the waveform reproduction and reduced the output error in the time domain. In the area of multiple shaking tables, Wang et al.^13^ has developed a series of new analytical models to study the control-structure interaction effects between a dual shaking table and the test structure, revealing the trends and the extent of the influence of the control-structure interaction on the model under different structural conditions. Li et al.^14^ analyzed the characteristics and patterns of the double shaking table-sample interaction on the system performance. The nonlinearity of the connection between the load and the table leads to the degradation of the system performance, manifested in the reduction of the waveform reproduction accuracy in the time domain and the appearance of resonance peaks in the frequency domain. It is necessary to propose compensation algorithms for eliminating the interaction forces generated by nonlinearity^15^. Li et al.^16^, to eliminate the influence of the interaction between load and table surface on the system control performance, introduced the force feedback compensation control based on three parameter control and carried out the experimental simulation analysis on the single-degree-of-freedom load and multi-degree-of-freedom load models in the single shaking table test. The results confirm that the feedback compensation control compensates for the interaction effect. The force transfer mechanism of a table-array system is more complex than a single shaking table. In addition to the interaction between the load and the table, there are also interactions between the shaking tables. Li et al.^12^ verified the effectiveness of the feedback compensation control algorithm in a double-shaking table test. However, this experiment's synchronized tracking performance of the table array system needs to be improved. To control the error, Li et al.^17^ proposed a differential motion synchronized tracking control (DMSTC) strategy to reduce the synchronization error and the maximum tracking error in differential motion. The load characteristics affect the interaction between the load and the table. Gao et al.^18^ considered a single shaking table system that modeled the load according to the spring-damping model and analyzed the effects of load mass, frequency, and damping on the control performance of a shaking table for earthquake simulation. To solve the impact of load characteristics on the response amplitude, a differential pressure feedback compensation algorithm is introduced based on modeling, which compensates for the interaction between the load and the table and improves the accuracy of waveform reproduction. Liang et al.^19^ proposed a feed-forward decoupling compensation control (FDCC) strategy to compensate for the effect of load characteristics on the system based on three-parameter control to realize wide-frequency excitation and high-precision control of a large-scale composite shaking table (LCST). The above study only considered the interaction between the load and the table, which can be simplified as the connection between the load and the table.

In practice, the shaking table system is affected by the connection between the load and the table and between the actuator and the table^10,20^. The intrinsic characteristics of most loads, tables, and shakers are quite different, i.e., the corresponding self-oscillating frequencies are in different ranges, and it is necessary to investigate whether there is a pattern between the two connection characteristics and the frequency response characteristics of the system. In addition, the conventional force feedback compensation requires deriving the interaction force equation^21^. Then the inverse compensation function, which is relatively complicated for the system considering both characteristics and the applicable compensation algorithms, needs to be explored. In this paper, based on the research on the influence of the connection characteristics of the load and the table, and the actuator and the table on the control performance of seismic simulation shaking table, then consider the influence of the two kinds of connection characteristics on the performance of the system at the same time, based on the results of the analysis, a flexible connection reaction force compensation algorithm is proposed.

## Method

### Analytic modeling

**Figure 1:**
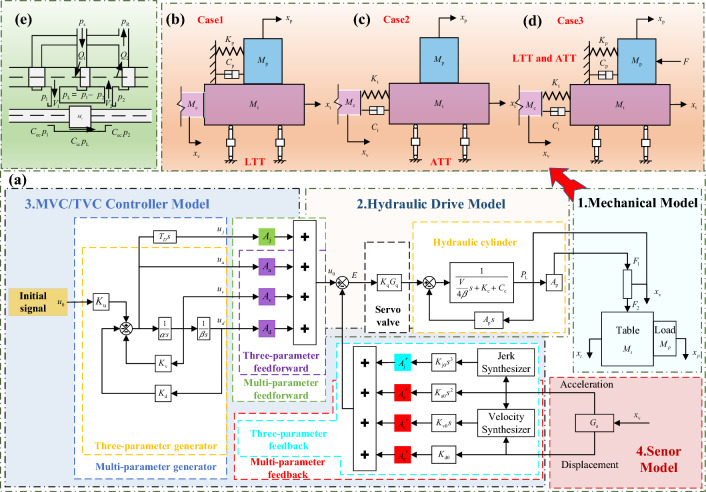
System Modeling. (**a**) Four sub-models of the system, (**b**) load and table (LTT) flexible connection model, (**c**) actuator and table (ATT) flexible connection model, (**d**) actuator, table, and load (LTT and ATT) flexible connection model, (**e**) hydraulic drive model.

A uniaxial shaking table is driven by a single horizontally oriented shaker with the load centered on the table surface and the actuating rod aligned with the center of the table surface. This allows a large degree of simulation of the effect of the flexible connection characteristics on the performance of the shaking table. Also, the derivation of the transfer function is simplified. The transfer function of the system is built according to the modular approach shown in Fig. 1. The method divides the shaking table and flexible connection characteristics into four important sub-models, including the mechanical model, the hydraulic drive model, the MVC/TVC controller model, and the sensor model, as shown in Fig. 1a. It is worth noting that the composition and physical properties of the four models are considered together in the modeling process. A detailed description of the notation is presented in the Appendix.

### Mechanical model

The mechanical model is the shaking table moving mechanism part and the connection property. The model of the shaking table considering the connection properties is shown in Fig. 1b–d. The detailed explanation of the symbols is as follows, $M_{c}$ is the mass of the actuator rod, $M_{t}$ is the mass of the table, $M_{p}$ is the mass of the load, $F_{1}$ is the excitation force of the hydraulic cylinder, $F_{2}$ is the excitation force of the actuator rod, $x_{v}$ is the displacement of the actuator rod, $x_{t}$ is the displacement of the table, $x_{p}$ is the displacement of the load, $K_{t}$ is the stiffness coefficient of the connection between the actuator rod and the table, $C_{t}$ is the damping coefficient of the connection between the actuator rod and the table, and $K_{p}$ is the stiffness coefficient of the connection between the load and the table, the $C_{p}$ is the damping coefficient of the connection between the load and the table.

Figure 1b shows the mechanical model of the shaking table considering the flexible connection of the load to the table (LTT). Figure 1c shows a mechanical model of a shaking table considering a flexible connection between the actuator and the table (ATT). Figure 1d shows a mechanical model of a shaking table considering a flexible connection between the actuator, table, and load (LTT and ATT). In Fig. 1b–d, the three mechanical models correspond to three working conditions (Case1, Case2 and Case3). In addition, the actuator, the table, and the load are considered as a complete whole, which is the equivalent model of the mechanical model.

Based on Fig. 1b–d, Newton’s second law was used to establish a mechanical model of the shaking table considering the flexible connection characteristics. The equivalent mechanical models for Case1 (LTT flexible connection), Case2 (ATT flexible connection), and Case3 (LTT and ATT flexible connection) are,

1 $$ \left\{ \begin{gathered} A_{p} p_{L} = M_{c} s^{2} x_{v} + M_{t} s^{2} x_{t} + (C_{p} s + K_{p} )(x_{t} - x_{p} ) \hfill \\ (C_{p} s + K_{p} )(x_{t} - x_{p} ) = M_{p} s^{2} x_{p} \hfill \\ x_{v} = x_{t} \hfill \\ \end{gathered} \right. $$

2 $$ \left\{ \begin{gathered} A_{p} p_{L} = M_{c} s^{2} x_{v} + (C_{t} s + K_{t} )(x_{v} - x_{t} ) \hfill \\ (C_{t} s + K_{t} )(x_{v} - x_{t} ) = M_{t} s^{2} x_{t} + M_{p} s^{2} x_{p} \hfill \\ x_{t} = x_{p} \hfill \\ \end{gathered} \right. $$

3 $$ \left\{ \begin{gathered} A_{p} p_{L} = M_{c} s^{2} x_{v} + (C_{t} s + K_{t} )(x_{v} - x_{t} ) \hfill \\ \hfill \\ (C_{t} s + K_{t} )(x_{v} - x_{t} ) = M_{t} s^{2} x_{t} + (C_{p} s + K_{p} )(x_{t} - x_{p} ) \hfill \\ \hfill \\ (C_{p} s + K_{p} )(x_{t} - x_{p} ) = M_{p} s^{2} x_{p} \hfill \\ \end{gathered} \right. $$

### Hydraulic drive modeling

The drive of the shaking table relies on a hydraulic drive system. The servo valve and the hydraulic cylinder are the two core parts of the hydraulic drive system, the schematic diagram of which is shown in Fig. 1e. Where the servo valve is considered as a second-order oscillating link with a transfer function:

4 $$ K_{q} = G_{q} k_{q} = \frac{{k_{q} }}{{\frac{{s^{2} }}{{n_{q}^{2} }} + \frac{{2D_{q} }}{{n_{q} }}s + 1}} $$

where $k_{q}$ is the flow gain of the servo valve, $n_{q}$ is the intrinsic frequency of the servo valve, and $D_{q}$ is the damping ratio of the servo valve.

Servo valves and hydraulic cylinders combined with the mechanical model constitute the hydraulic three-continuum equation, and simplified the form of the conversion can be described as follows:

Case1 (LTT flexible connection):

5 $$ \left\{ \begin{gathered} (M_{c} + M_{t} + M_{p} \varphi_{1} )s^{2} x_{v} = A_{p} p_{L} \hfill \\ Q_{L1} = A_{p} sx_{v} + \frac{V}{4\beta }sp_{L} + C_{c} p_{L} \hfill \\ Q_{L2} = K_{q} E - K_{c} p_{L} \hfill \\ \end{gathered} \right. $$

where $\varphi_{1}$ is the transfer function of load displacement and table displacement under Case1. Its expressive form is:

6 $$ \varphi_{1} = \frac{{x_{p} }}{{x_{t} }} = \frac{{C_{p} s + K_{p} }}{{M_{p} s^{2} + C_{p} s + K_{p} }} = \frac{{2\xi_{p} \omega_{p} s + \omega_{p}^{2} }}{{s^{2} + 2\xi_{p} \omega_{p} s + \omega_{p}^{2} }} $$

where $\xi_{p}$, $\omega_{p}$ are the damping ratio and intrinsic frequency of Case1 (LTT flexible connection).

Case2 (ATT flexible connection):

7 $$ \left\{ \begin{gathered} [M_{c} + (M_{t} + M_{p} )\varphi_{2} ]s^{2} x_{v} = A_{p} p_{L} \hfill \\ Q_{L1} = A_{p} sx_{v} + \frac{V}{4\beta }sp_{L} + C_{c} p_{L} \hfill \\ Q_{L2} = K_{q} E - K_{c} p_{L} \hfill \\ \end{gathered} \right. $$

where $\varphi_{2}$ is the transfer function of table displacement and actuator displacement under Case2. Its representation is in the form:

8 $$ \varphi_{2} = \frac{{x_{t} }}{{x_{v} }} = \frac{{C_{t} s + K_{t} }}{{(M_{t} + M_{p} )s^{2} + C_{t} s + K_{t} }} = \frac{{2\xi_{t} \omega_{t} s + \omega_{t}^{2} }}{{s^{2} + 2\xi_{t} \omega_{t} s + \omega_{t}^{2} }} $$

where $\xi_{t}$, $\omega_{t}$ are the damping ratio and intrinsic frequency of Case2 (ATT flexible connection).

Case3 (LTT and ATT flexible connection):

9 $$ \left\{ \begin{gathered} (M_{c} + M_{t} \varphi_{4} + M_{p} \varphi_{3} \varphi_{4} )s^{2} x_{v} = A_{p} p_{L} \hfill \\ Q_{L1} = A_{p} sx_{v} + \frac{V}{4\beta }sp_{L} + C_{c} p_{L} \hfill \\ Q_{L2} = K_{q} E - K_{c} p_{L} \hfill \\ \end{gathered} \right. $$

where $\varphi_{3}$ is the transfer function of load displacement and table displacement under Case3, $\varphi_{4}$ is the transfer function of table displacement and actuator displacement under Case3. The representations of $\varphi_{3}$ and $\varphi_{4}$ are:

10 $$ \varphi_{3} = \frac{{x_{p} }}{{x_{t} }} = \frac{{C_{p} s + K_{p} }}{{M_{p} s^{2} + C_{p} s + K_{p} }} = \frac{{2\xi_{p1} \omega_{p1} s + \omega_{p1}^{2} }}{{s^{2} + 2\xi_{p1} \omega_{p1} s + \omega_{p1}^{2} }} $$

11 $$ \varphi_{4} = \frac{{x_{t} }}{{x_{v} }} = \frac{{C_{t} s + K_{t} }}{{(M_{t} + M_{p} \varphi_{1} )s^{2} + C_{t} s + K_{t} }} = \frac{{2\xi_{t1} \omega_{t1} s + \omega_{t1}^{2} }}{{s^{2} + 2\xi_{t1} \omega_{t1} s + \omega_{t1}^{2} }} $$

where $\xi_{p1}$ and $\omega_{p1}$ are the damping ratio and intrinsic frequency of the load to the table (LTT flexible connection), $\xi_{t1}$ and $\omega_{t1}$ are the damping ratio and intrinsic frequency of the actuator to the table (ATT flexible connection).

### MVC/TVC controller model

The MVC/TVC controller model is the control part of the shaking table. The MVC/TVC controller model consists of a feedback loop, a feed-forward loop, and a generator, and its schematic diagram is shown in Fig. 1a. Compared with the three-parameter control, the multiparameter management introduces a set of feedforward feedback control variables (Jerk) on top of the three feedforward feedback control variables (displacement, velocity, acceleration). According to the TVC system modeling procedure given in Ref. 40, the control error signal is:

12 $$ E = G_{\alpha } u_{0} - G_{\beta } G_{a} x_{v} $$

where $E$ is the control error signal, $u_{0}$ is the control signal, $G_{\alpha }$ is the MVC/TVC generator and feedforward transfer function, $G_{\beta }$ is the MVC/TVC feedback transfer function, and $G_{a}$ is the sensor transfer function.

### Sensor model

In general, the sensor can be viewed as a second-order oscillating link, and the transfer function of the sensor is:

13 $$ G_{a} = \frac{1}{{\frac{{s^{2} }}{{n_{a}^{2} }} + \frac{{2D_{a} }}{{n_{a} }}s + 1}} $$

where $n_{a}$ is the intrinsic frequency of the sensor and $D_{a}$ is the damping ratio of the sensor.

### Transfer function modeling

The transfer function of the system is built based on the above four models, which can be described as:

14 $$ \left\{ \begin{gathered} \frac{x}{u} = \frac{1}{{K_{d0} A_{d}^{\prime } G_{a} G_{\alpha } }}\frac{1}{{B_{2} + B_{3} }} \hfill \\ B_{2} = \frac{{K_{a0} A_{j}^{\prime } }}{{K_{d0} A_{d}^{\prime } }}s^{2} + \frac{{K_{v0} A_{j}^{\prime } }}{{K_{d0} A_{d}^{\prime } }}s + 1 \hfill \\ B_{3} = \frac{1}{{K_{q} G_{a} }}\left( {\frac{{M_{Ei} V}}{{4\beta A_{p}^{2} }}s^{3} + \frac{{M_{Ei} (K_{c} + C_{c} )}}{{A_{p}^{2} }}s^{2} + s} \right) \hfill \\ \end{gathered} \right.OR\left\{ \begin{gathered} \frac{x}{u} = \frac{1}{{K_{d0} A_{d}^{\prime } G_{a} G_{\alpha } }}\frac{1}{{B_{2} + B_{3} }} \hfill \\ B_{2} = \frac{{K_{j0} A_{j}^{\prime } }}{{K_{d0} A_{d}^{\prime } }}s^{3} + \frac{{K_{a0} A_{j}^{\prime } }}{{K_{d0} A_{d}^{\prime } }}s^{2} + \frac{{K_{v0} A_{j}^{\prime } }}{{K_{d0} A_{d}^{\prime } }}s + 1 \hfill \\ B_{3} = \frac{1}{{K_{q} G_{a} }}\left( {\frac{{M_{Ei} V}}{{4\beta A_{p}^{2} }}s^{3} + \frac{{M_{Ei} (K_{c} + C_{c} )}}{{A_{p}^{2} }}s^{2} + s} \right) \hfill \\ \end{gathered} \right. $$

where $M_{Ei}$ is the equivalent mass and $i$ is the Case labeling, $K_{d0}$, $K_{v0}$, $K_{a0}$, and $K_{j0}$ are the displacement feedback normalized sensitivity, the velocity feedback normalized sensitivity, the acceleration feedback normalized sensitivity coefficient, $A_{d}^{\prime }$, $A_{v}^{\prime }$, $A_{a}^{\prime }$, and $A_{j}^{\prime }$ are displacement feedback gain, velocity feedback gain, acceleration feedback gain, and jerk feedback gain, respectively.

The expressions for the equivalent masses $M_{E1}$, $M_{E2}$ and $M_{E3}$ are given below:

15 $$ \left\{ \begin{gathered} M_{E1} = M_{c} + M_{t} + M_{p} \varphi_{1} \hfill \\ M_{E2} = M_{c} + (M_{t} + M_{p} )\varphi_{2} \hfill \\ M_{E3} = M_{e} + M_{t} \varphi_{4} + M_{p} \varphi_{3} \varphi_{4} \hfill \\ \end{gathered} \right. $$

### Algorithm for compensation of reaction forces in flexible connections

**Figure 2:**
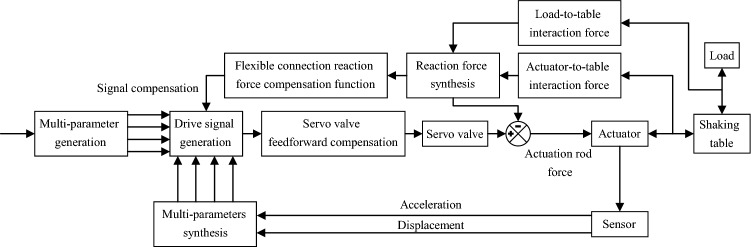
Flow chart of reaction force feedback compensation.

From the theoretical analysis above, it is clear that the flexible connection can affect the performance of the shaking table, critically because of the interaction forces between the actuator, the table, and the load. Therefore, compensation for shaking table performance can be achieved by simply eliminating these interacting forces. Based on the model of Fig. 1, the compensation algorithm is added and the schematic diagram of compensation is shown in Fig. 2. The principle of operation is that the counterforce value is calculated from the transfer function between the load, shaking table, and actuator, and signal compensation is achieved by flexibly connecting the counterforce compensation function. It should be noted that in this study, the sensor measures the acceleration and displacement of the actuator.

**Figure 3:**
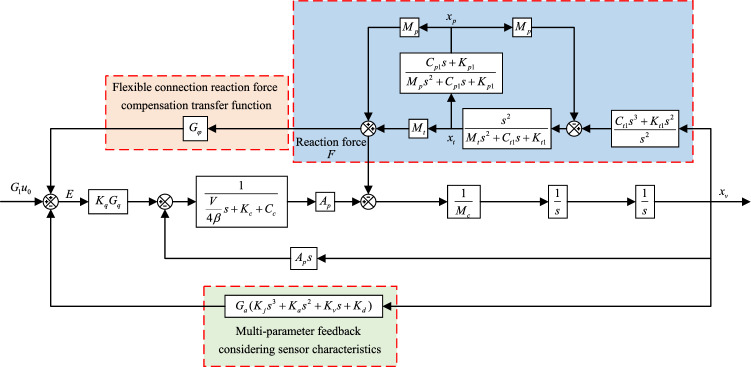
Schematic diagram of the reaction force compensation algorithm for flexible connections.

Based on Fig. 1 combined with the compensation algorithm a block diagram of the transfer function is obtained (e.g. Fig. 3).

**Figure 4:**
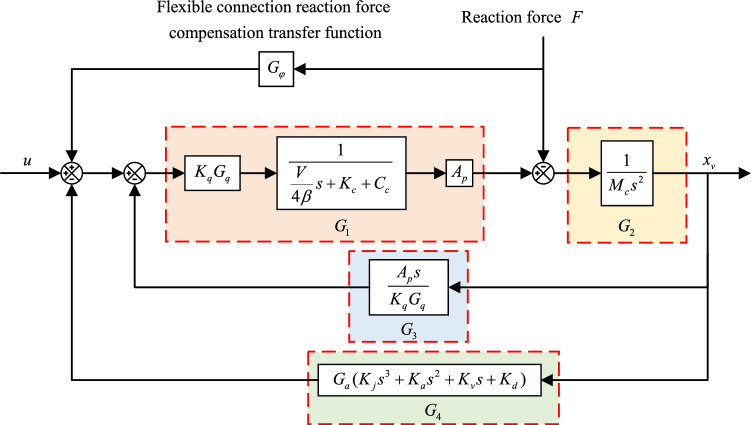
Simplified schematic diagram of the reaction force compensation algorithm for flexible connections.

In Fig. 3 the compensation function is the core of the compensation algorithm, and Fig. 3 is simplified to Fig. 4 for ease of derivation. After adding the compensation algorithm, the output expression of the system is:

16 $$ x_{v} = \frac{{G_{1} G_{2} }}{{1 + G_{2} G_{3} + G_{2} G_{4} }}u + \frac{{(G_{1} G_{\varphi } - 1)G_{2} }}{{1 + G_{2} G_{3} + G_{2} G_{4} }}F $$

Making the coefficients of Eq. (16) containing the F term zero yields the inverse transfer function of the compensation algorithm (compensation transfer function):

17 $$ G_{\varphi } = \frac{1}{{G_{1} }} = \frac{{K_{c} + C_{c} + \frac{V}{4\beta }s}}{{K_{q} A_{p} }} $$

In the real system, it is not possible to use the differential link, a turning frequency must be introduced to improve this, the selection of the turning frequency is required to be higher than the overall frequency of the system, from which a new compensated transfer function is obtained:

18 $$ G_{\varphi } = \frac{{K_{c} + C_{c} + \frac{V}{4\beta }s}}{{K_{q} A_{p} \left( {\frac{s}{2\pi f} + 1} \right)}} $$

## Results and discussion

### Open-loop system

Based on the transfer function, the sensitivity of the intrinsic frequency, damping ratio, and load mass to the transfer function is analyzed in depth for different case models, as well as the trend and extent of the effect on the transfer function for different connection characteristics.

The analysis is carried out at different intrinsic frequencies, damping ratios, and load masses. According to Tables in the Supplementary Material, different intrinsic frequencies, damping ratios, and load masses are taken for considering individual connection characteristics as detailed in Table 1. In Table 1, $\omega_{p}$ is denoted as $\omega_{p} = \sqrt {{{K_{p} } \mathord{\left/ {\vphantom {{K_{p} } {M_{p} }}} \right. \kern-0pt} {M_{p} }}}$, $\xi_{p}$ is denoted as $\xi_{p} = {{C_{p} } \mathord{\left/ {\vphantom {{C_{p} } {\left( {2M_{p} \omega_{p} } \right)}}} \right. \kern-0pt} {\left( {2M_{p} \omega_{p} } \right)}}$, $\omega_{t}$ is denoted as $\omega_{t} = \sqrt {{{K_{t} } \mathord{\left/ {\vphantom {{K_{t} } {(M_{t} + M_{p} )}}} \right. \kern-0pt} {(M_{t} + M_{p} )}}}$, $\xi_{t}$ is denoted as $\xi_{t} = {{C_{t} } \mathord{\left/ {\vphantom {{C_{t} } {\left[ {2\left( {M_{t} + M_{p} } \right)\omega_{t} } \right]}}} \right. \kern-0pt} {\left[ {2\left( {M_{t} + M_{p} } \right)\omega_{t} } \right]}}$. According to the assumed conditions, the values of different intrinsic frequencies, damping ratios, and load masses set to consider the two flexible connections are detailed in Table 2. The expressions all contain complex transfer functions, so approximations are taken. In order to compare and analyze the influence of the connection characteristics on the transfer function, the shaking table no-load condition was defined as a reference. The main point to note is that the amplitude-frequency characteristics of the system, as a measure of the performance of the shaking table, are considered to be in the effective operating range within ± 3 dB.

**Table 1:** Different intrinsic frequencies, damping ratios and load masses under LTT or ATT flexible connection.

Flexible connection type	Conditions	$M_{p}$ (kg)	$\omega_{p}$	$\xi_{p}$	$\omega_{t}$	$\xi_{t}$
LTT	Different inherent frequency conditions	10,000	3 Hz	0.02	–	–
		10,000	6 Hz	0.02	–	–
		10,000	9 Hz	0.02	–	–
	Different damping ratio conditions	10,000	6 Hz	0.02	–	–
		10,000	6 Hz	0.04	–	–
		10,000	6 Hz	0.08	–	–
	Different load mass conditions	5000	6 Hz	0.02	–	–
		10,000	6 Hz	0.02	–	–
		15,000	6 Hz	0.02	–	–
ATT	Different inherent frequency conditions	10,000	–	–	3 Hz	0.02
		10,000	–	–	6 Hz	0.02
		10,000	–	–	9 Hz	0.02
	Different damping ratio conditions	10,000	–	–	6 Hz	0.02
		10,000	–	–	6 Hz	0.04
		10,000	–	–	6 Hz	0.08
	Different load mass conditions	5000	–	–	6 Hz	0.02
		10,000	–	–	6 Hz	0.02
		15,000	–	–	6 Hz	0.02

**Table 2:** Different intrinsic frequencies, damping ratios and load masses under LTT and ATT flexible connection.

Flexible connection type	Conditions	$M_{p}$ (kg)	$\omega_{p}$ (Hz)	$\xi_{p}$	$\omega_{t}$ (Hz)	$\xi_{t}$
LTT and ATT	Different inherent frequency conditions (LTT)	10,000	3	0.02	6	0.02
		10,000	6	0.02	6	0.02
		10,000	9	0.02	6	0.02
	Different damping ratio conditions (LTT)	10,000	6	0.02	6	0.02
		10,000	6	0.04	6	0.02
		10,000	6	0.08	6	0.02
	Different inherent frequency conditions (ATT)	10,000	6	0.02	3	0.02
		10,000	6	0.02	6	0.02
		10,000	6	0.02	9	0.02
	Different damping ratio conditions (ATT)	10,000	6	0.02	6	0.02
		10,000	6	0.02	6	0.04
		10,000	6	0.02	6	0.08
	Different load mass conditions	5000	6	0.02	6	0.02
		10,000	6	0.02	6	0.02
		15,000	6	0.02	6	0.02

**Figure 5:**
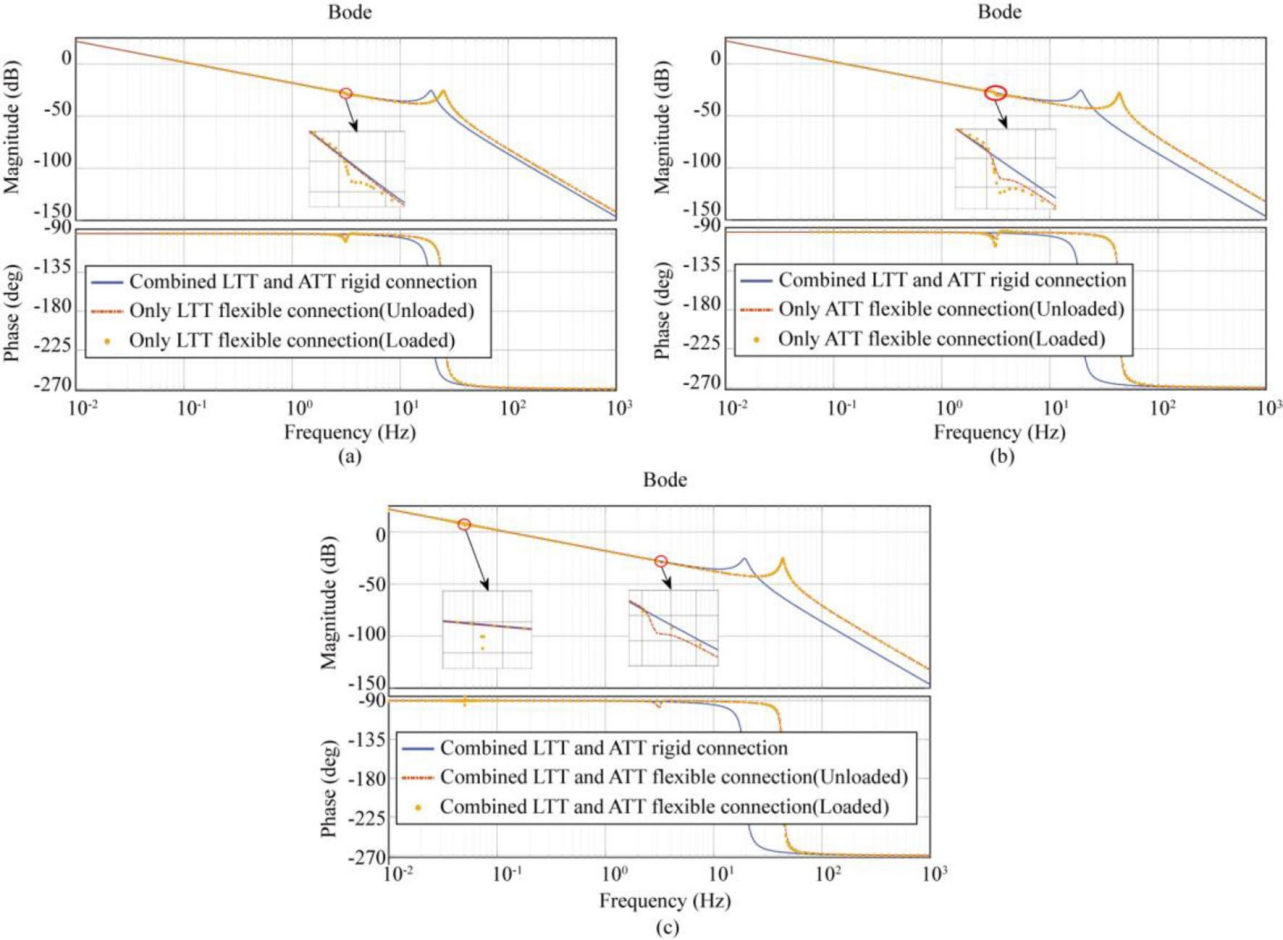
Effect of load and flexible connection on transfer function. (**a**) Case1 (LTT flexible connection), (**b**) Case2 (ATT flexible connection), (**c**) Case3 (LTT and ATT flexible connection).

Figure 5a shows that the oil column resonance peak appears at 19.39 Hz with an amplitude of − 25.34 dB for a completely rigid connection, at 25.07 Hz with an amplitude of − 25.36 dB under the action of no load and LTT flexible connection, and at 25.20 Hz with an amplitude of − 26.01 dB under the action of load and LTT flexible connection. Figure 5a shows that the oil column resonance peak appears at 19.39 Hz with an amplitude of − 25.34 dB for a fully rigid connection, at 25.07 Hz with an amplitude of − 25.36 dB for no load and LTT flexible connection, and at 25.20 Hz with an amplitude of − 26.01 dB for a load and LTT flexible connection. In addition, to load and LTT flexible connection, the resonance peak of the open-loop system appears at 3.25 Hz, with an amplitude of − 29.31 dB. From the above data, it can be seen that the oil column resonance peak is delayed by considering the LTT flexible connection, and the resonance peak can only be generated by the combination of the load and the LTT flexible connection.

Figure 5b shows that the oil column resonance peak appears at 19.39 Hz with an amplitude of − 25.34 dB for a fully rigid connection, at 43.71 Hz with an amplitude of − 27.15 dB for no load and ATT flexible connection, and at 43.94 Hz with an amplitude of − 28.65 dB for a load and ATT flexible connection; furthermore, the resonance peak appears at 3.25 Hz for the open-loop system with an amplitude of − 29.31 dB for a load and ATT flexible connection; from the above data, it is clear that the oil column resonance peak appears at 3.25 Hz for the open-loop system considering the LTT flexible connection, and the amplitude is − 29.31 dB and ATT flexible connection, the resonance peak of the open-loop system appears at 3.24 Hz, with an amplitude of − 29.39 dB; and the resonance peak of the open-loop system under load and ATT flexible connection appears at 3.25 Hz, with an amplitude of − 30.37 dB. From the above data, it can be seen that, considering that the ATT flexible connection delays the emergence of the resonance peak of the oil column; and that either no-load or load with ATT flexible connection can generate the resonance peak. The resonance peak and the load increase the amplitude, which is due to the fact that the sum of the mass of the table and the load in Case 2 determines the magnitude of the interaction force between the actuator and the table.

Figure 5c shows that the oil column resonance peak appears at 19.39 Hz with an amplitude of − 25.34 dB for the fully rigid connection; the oil column resonance peak appears at 43.71 Hz with an amplitude of − 27.15 dB for the no-load, LTT, and ATT flexible connection; the oil column resonance peak appears at 43.47 Hz with an amplitude of − 25.38 dB for the load, LTT, and ATT flexible connection; in addition, the open-loop system appears at 3.25 Hz with an amplitude of − 29.40 dB for the load and ATT flexible connection. In addition, under no load, LTT and ATT flexible connection, the resonance peak of the open-loop system occurs at 3.25 Hz with a magnitude of − 29.40 dB; under load, LTT and ATT flexible connection, the resonance peak of the open-loop system occurs at 0.05 Hz with a magnitude of 6.28 dB. From the above data, it can be seen that the consideration of the two flexible connections causes the resonance peak of the oil column to appear after a delay; the interaction of the reaction forces generated between the two flexible connections results in the resonance peak appearing in the low-frequency band.

### Analysis of joint actions considering connection characteristics under three-parametric and multi-parametric controls

**Figure 6:**
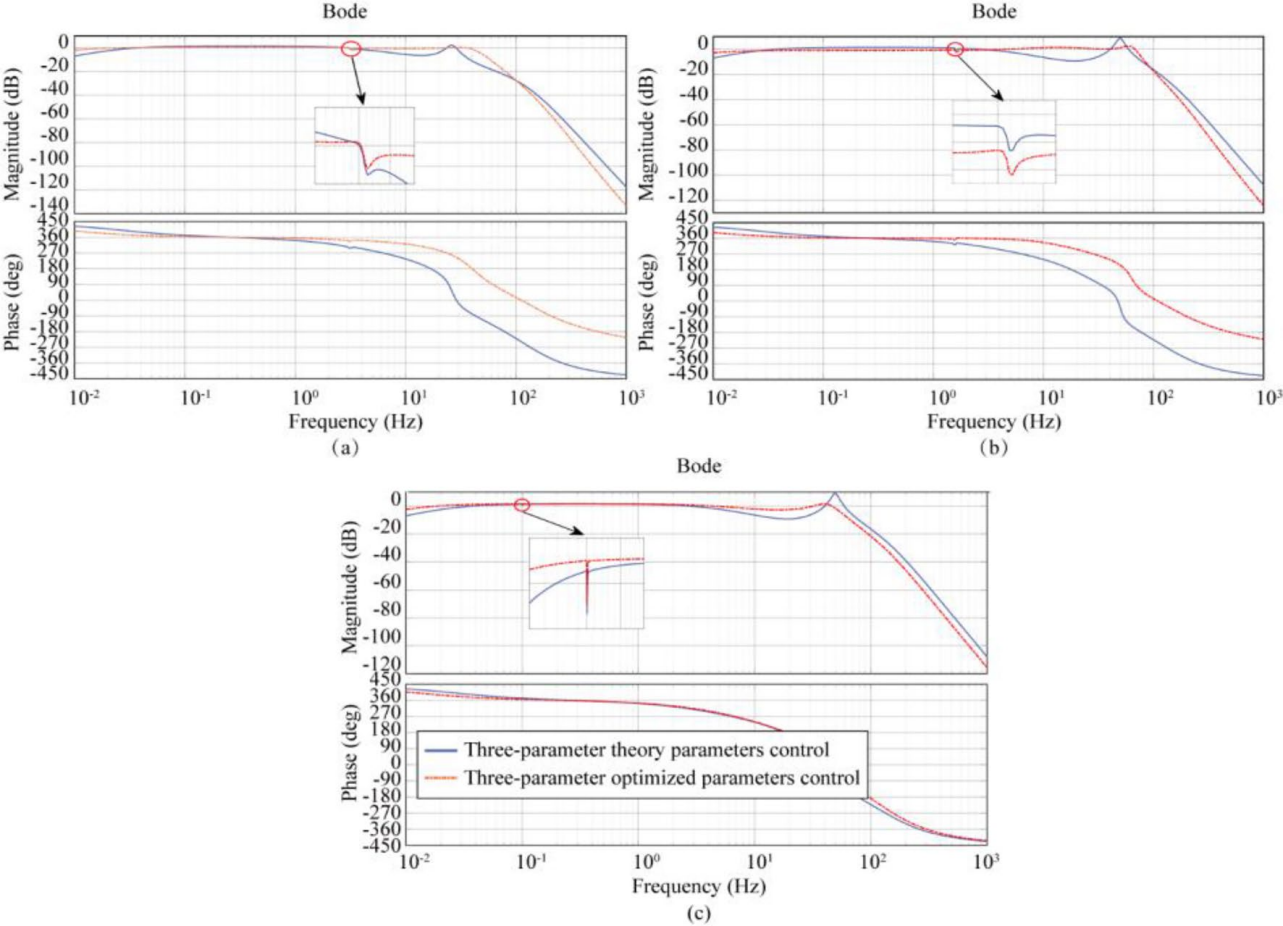
Frequency response characteristic curves of the system considering different connection characteristics under the three-parameter theory and optimization parameters: (**a**) LTT flexible connection, (**b**) ATT flexible connection, (**c**) LTT and ATT flexible connection.

**Figure 7:**
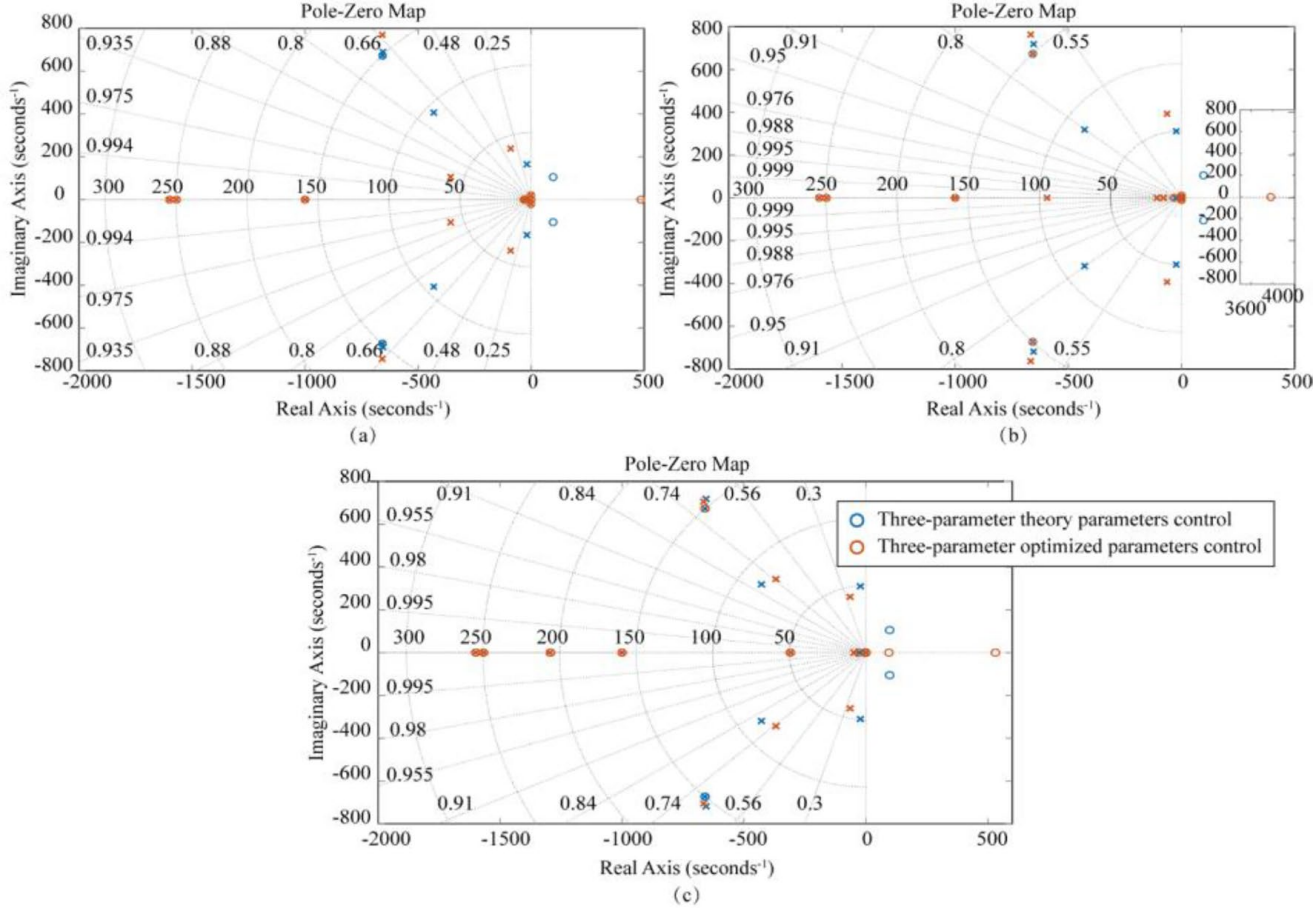
Zero-polar coordinates of the system considering different connection characteristics under the three-parameter theory and optimization parameters: (**a**) LTT flexible connection, (**b**) ATT flexible connection, (**c**) LTT and ATT flexible connection. (“ × ” stands for pole, and “○” stands for zero point).

After theoretical validation and simulation analysis, the values of each parameter under three-variable control (TVC) are selected, as shown in Table 3. Figure 6 shows the amplitude-frequency characteristics of Case1 (LTT flexible connection), Case2 (ATT flexible connection), and Case3 (LTT and ATT flexible connection) and Fig. 7 shows the polar plot of the system.

**Table 3:** Selected values of each parameter under three-parameter control.

Parameter name	Theoretical parameters	Optimization parameters		
	Case1-3	Case1	Case2	Case3
$A_{d}$	0.9969	0.35	0.30	0.5
$A_{v}$	0.0604	− 0.073	− 0.060	0.04
$A_{a}$	0.0097	− 0.005	− 0.0005	0.002
$A_{d}^{\prime }$	0.9969	0.35	0.30	0.50
$A_{v}^{\prime }$	0	− 0.004	0.20	0.15
$A_{a}^{\prime }$	0.0159	0.085	0.035	0.007

In Fig. 6, the three-parameter theoretical control (TTC) is unable to achieve a bandwidth of 0.1–0.3 Hz, but the three-parameter optimal control (TOC) can meet this requirement. After using the optimized parameters, the Case1 (LTT flexible connection) model bandwidth was expanded to 0.0080–45.0 Hz, and the amplitude of the oil column resonance peak corresponding to 26.13 Hz was reduced from 2.24 to 0.12 dB; The frequency bandwidth of the Case2 (ATT flexible connection) model was expanded to 0.0096–72.6 Hz, and the amplitude of the oil column resonance peak corresponding to 49.46 Hz was reduced from 8.80 to 1.14 dB; Case3 (LTT and ATT flexible connection) model bandwidth is expanded to 0.0091–52.3 Hz, and the amplitude of the oil column resonance peak at 49.26 Hz corresponds to a decrease from 9.04 to − 0.87 dB. From the above data, it can be seen that the three-parameter optimal control (TOC) broadens the effective bandwidth and eliminates the oil column resonance peaks. In Fig. 7, the poles of Case1, Case2, and Case3 models all appear in the left plane, side by side demonstrating the stability of the three-parameter optimal control (TOC).

**Figure 8:**
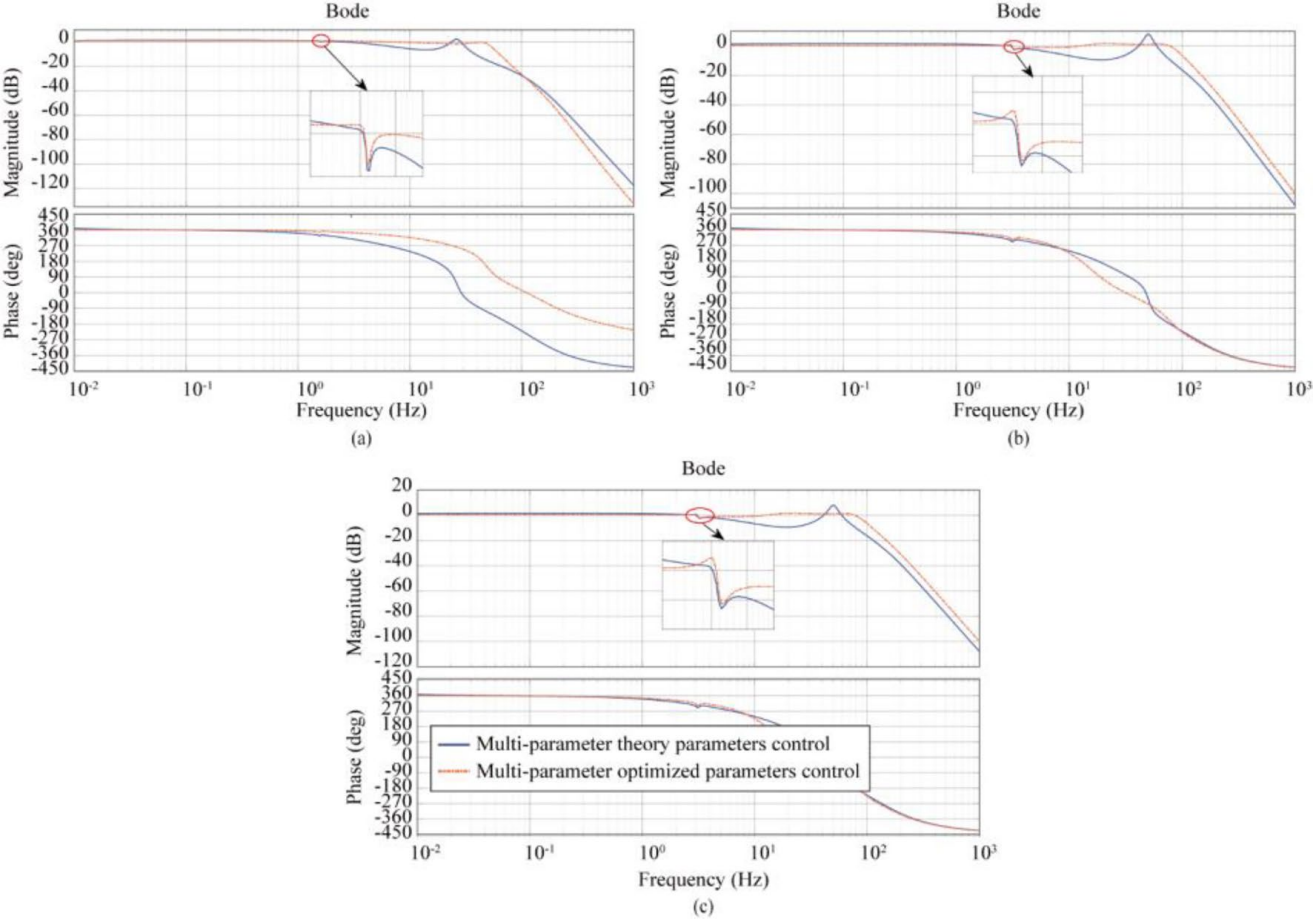
Frequency response characteristic curves of the system considering different connection characteristics under multi-parameter theory and optimization parameters: (**a**) LTT flexible connection, (**b**) ATT flexible connection, (**c**) LTT and ATT flexible connection.

**Figure 9:**
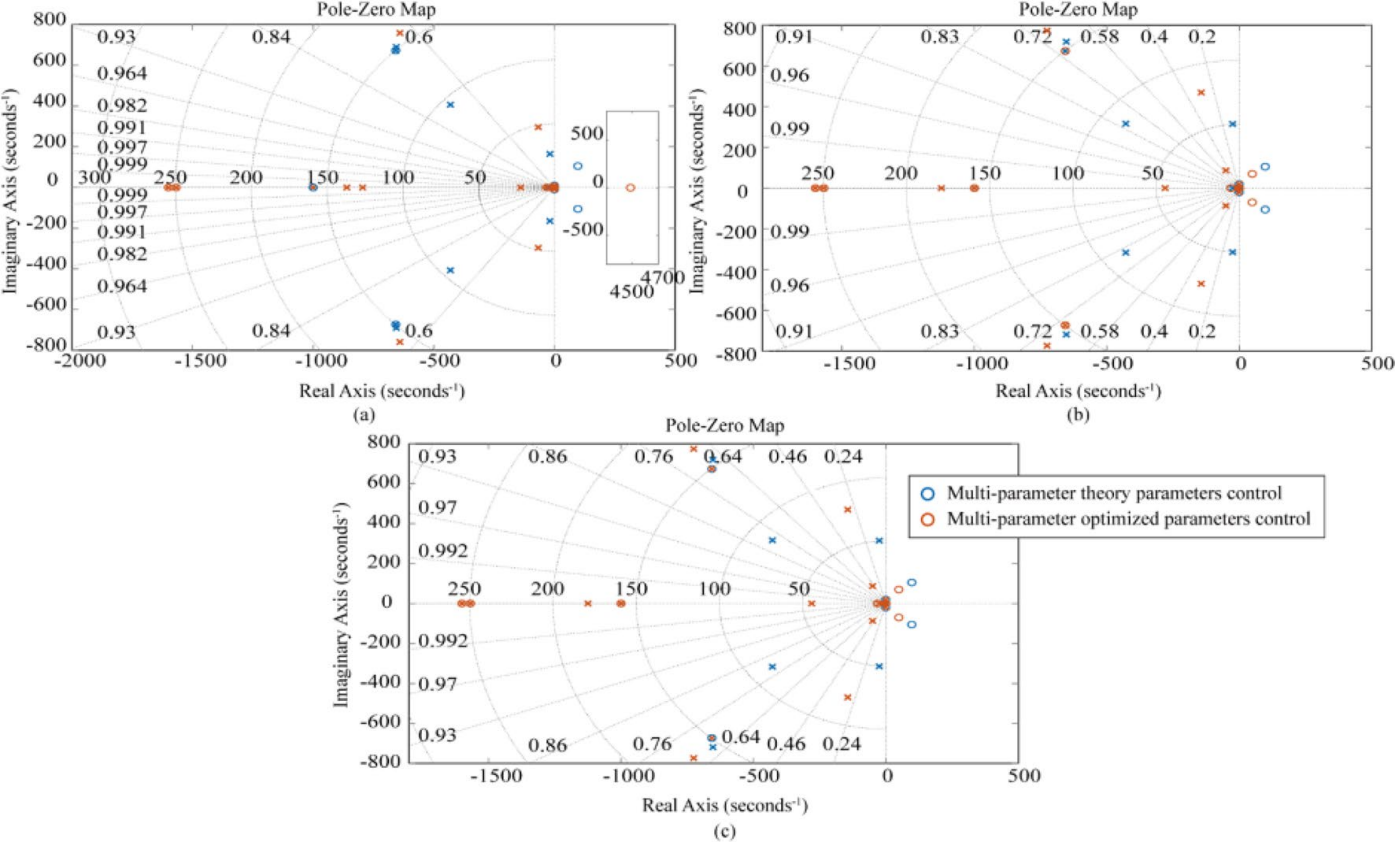
Zero-pole coordinates of the system considering different connection characteristics with multi-parameter theory and optimization parameters: (**a**) only LTT flexible connection, (**b**) only ATT flexible connection, (**c**) LTT and ATT flexible connection.

After theoretical validation and simulation analysis, the values of each parameter under multi-variable control (MVC) were selected as shown in Table 4. Figure 8 shows the amplitude-frequency characteristics for Case 1 (LTT flexible connection), Case 2 (ATT flexible connection), and Case 3 (LTT and ATT flexible connection), and Fig. 9 shows the polar plot of the system.

**Table 4:** Selected values of each parameter under multi-parameter control.

Parameter name	Theoretical parameters	Optimization parameters		
	Case1-3	Case1	Case2	Case3
$A_{d}$	0.9969	0.35	0.35	0.35
$A_{v}$	0.0604	− 0.073	− 0.03	0.03
$A_{a}$	0.0097	− 0.005	− 0.0097	0.0097
$A_{j}$	0	0	0	0
$A_{d}^{\prime }$	0.9969	0.35	0.35	0.35
$A_{v}^{\prime }$	0	− 0.004	0.35	0.35
$A_{a}^{\prime }$	0.0159	0.085	0.05	0.05
$A_{j}^{\prime }$	0.00018	0.06	− 0.065	− 0.065

In Fig. 8, there is little difference between the control performance of multi-parametric theoretical control (MTC) and three-parametric theoretical control (TTC), but the multi-parametric optimal control (MOC) can further extend the control performance based on the three-parametric optimal control (TOC). After using the optimized parameters, the bandwidth of the model for Case 1 (LTT flexible connection) is extended to 0.0011–52.2 Hz, and the amplitude of the oil column resonance peak corresponding to 26.05 Hz is reduced from 2.24 to − 1.46 dB; The frequency bandwidth of the model for Case 2 (ATT flexible connection) is extended to 0. 0011–89.10 Hz, and the peak amplitude of the corresponding oil column resonance at 49.91 Hz is reduced from 8.03 to 1.12 dB; The bandwidth of the model for case 3 (LTT and ATT flexible connection) was expanded to 0.0011–89.0 Hz, and the peak amplitude of the corresponding oil column resonance at 49.91 Hz was reduced from 7.97 to 1.06 dB. From the above data, it can be seen that the Multiparameter Optimal Control (MOC) is superior to the Three Covariate Optimal Control (TOC). In Fig. 9, the poles of Case 1, Case 2 and Case 3 models appear in the left plane, showing the stability of Multiparameter Optimal Control (MOC).

### Effect of different flexible connection characteristics on performance

Despite the stability brought to the system by the addition of the MTV/TVC control, the laws governing the effect of different flexible connection metrics on the performance of the shaking table deserve to be further explored. The Case1 (LTT flexible connection) model uses data from Tables 1 and 4.

**Figure 10:**
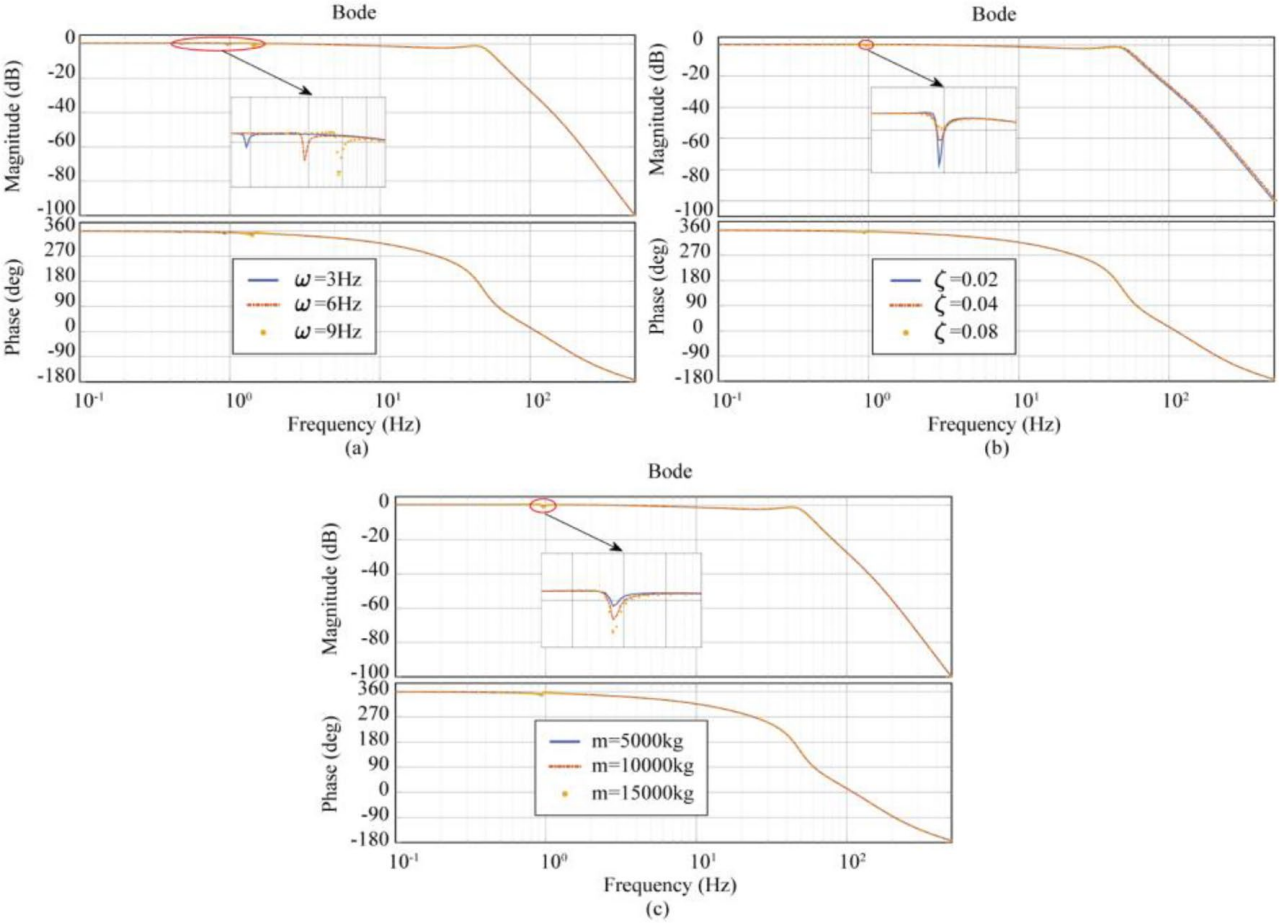
Effect of LTT flexible connection characteristics on transfer function. (**a**) Effect of intrinsic frequency on transfer function, (**b**) effect of damping ratio on transfer function, (**c**) effect of load mass on transfer function.

Figure 10 shows the effect of different intrinsic frequencies, damping ratios, and load mass on the transfer function of the Case1 (LTT flexible connection) model. Figure 10a shows that as the LTT flexible connection intrinsic frequency increases from 3 to 9 Hz, the resonance peak frequency increases from 0.48 to 1.43 Hz, and the amplitude corresponding to peaks and valleys decreases from − 0.21 to − 1.46 dB. Figure 10b shows that as the LTT flexible connection damping ratio increases from 0.02 to 0.08, the resonance peak frequency increases from 0.95 to 0.97 Hz, and the amplitude corresponding to the peaks and valleys increases from − 0.82 to 0.05 dB. Figure 10c shows that as the load mass increases from 5000 to 15,000 kg, the resonance peak frequencies are all 0.95 Hz, and the amplitude corresponding to the peaks and valleys decreases from − 0.24 to − 1.36 dB. Based on these data, it can be concluded that the degree of influence on shaking table performance is positively related to the intrinsic frequency of the LTT flexible connection, negatively related to the damping ratio of the LTT flexible connection, and positively related to the load mass; The frequencies corresponding to the resonance peaks are positively correlated with the intrinsic frequency of the LTT flexible connection, and not significantly correlated with the damping ratio and load mass of the LTT flexible connection; The resonance peak amplitude is positively correlated with the intrinsic frequency of the LTT flexible connection, negatively correlated with the damping ratio of the LTT flexible connection, and positively correlated with the load mass.

**Figure 11:**
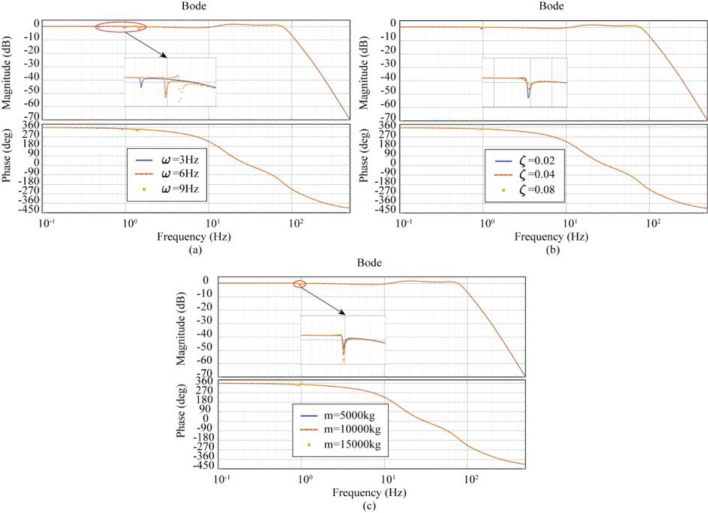
Effect of ATT flexible connection characteristics on transfer function. (**a**) Effect of intrinsic frequency on transfer function, (**b**) effect of damping ratio on transfer function, (**c**) effect of load mass on transfer function.

The Case2 (ATT Flexible Connection) model uses the data in Tables 1 and 4. Figure 11 shows the effect of different intrinsic frequencies, damping ratios, and load mass on the transfer function of the model for Case 2 (ATT flexible connection). Figure 11a shows that as the intrinsic frequency of the ATT flexible connection increases from 3 to 9 Hz, the peak resonance frequency increases from 0.48 to 1.42 Hz, and the amplitude corresponding to the peaks and valleys decreases from − 0.47 to − 1.57 dB. Figure 11b shows that as the ATT flexible connection damping ratio increases from 0.02 to 0.08, the peak resonance frequency increases from 0.95 to 0.98 Hz, and the amplitude corresponding to the peaks and valleys decreases from − 1.28 to − 0.16 dB. Figure 11c shows that as the load mass increases from 5000 to 15000 kg, the peak resonance frequency decreases from 0.95 Hz and the corresponding amplitude of the valley decreases from − 0.75 to − 1.78 dB. Based on these data, it can be concluded that the degree of influence on shaking table performance is positively related to the intrinsic frequency of the ATT flexible connection, negatively related to the damping ratio of the ATT flexible connection, and positively related to the load mass; The frequencies corresponding to the resonance peaks are positively correlated with the intrinsic frequency of the ATT flexible connection and are not significantly correlated with the damping ratio and load mass of the ATT flexible connection; The resonance peak amplitude is positively correlated with the intrinsic frequency of the ATT flexible connection, negatively correlated with the damping ratio of the ATT flexible connection, and positively correlated with the load mass.

**Figure 12:**
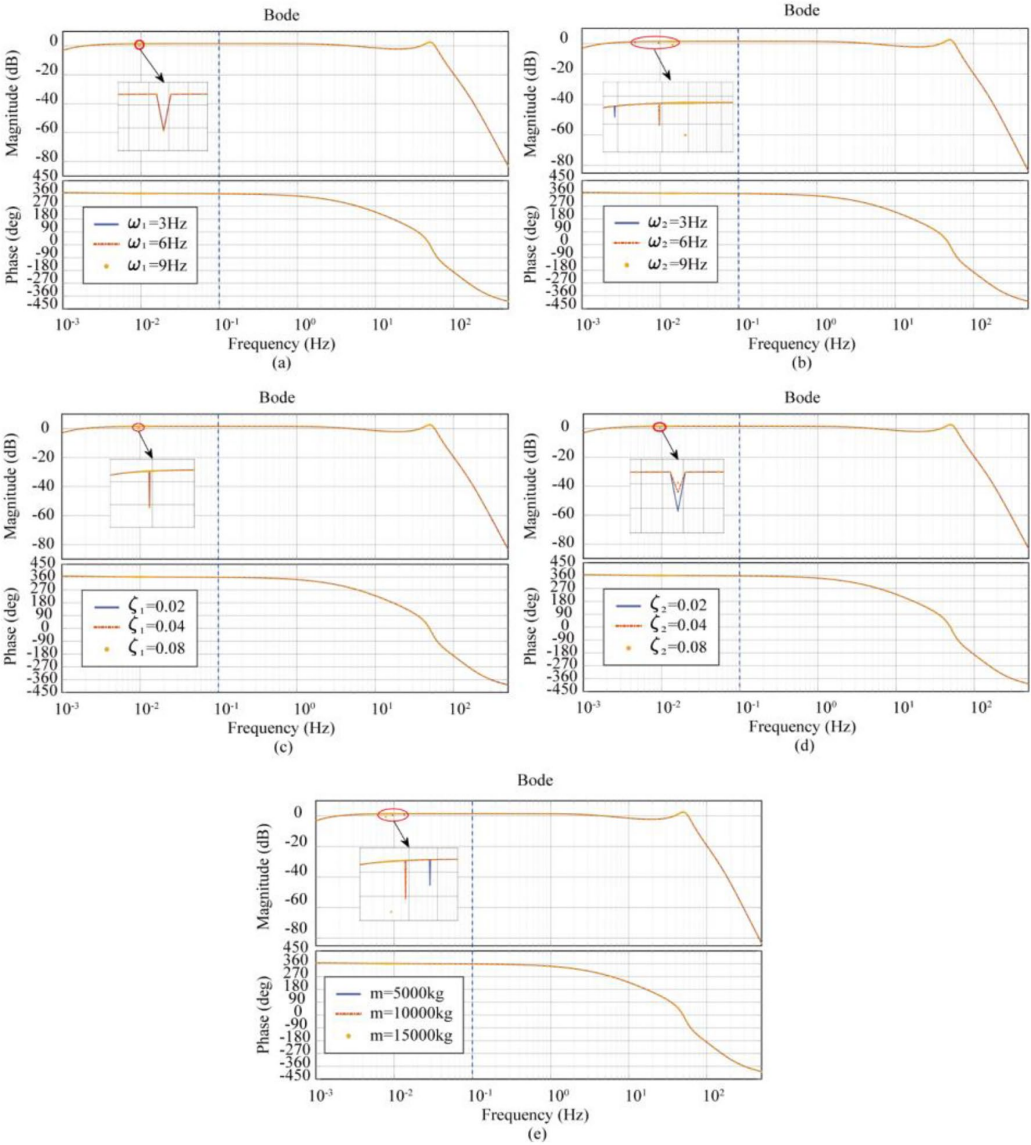
Effect of LTT and ATT flexible connection characteristics on the transfer function (**a**) effect of LTT flexible connection’s $\omega_{1}$ on the transfer function, (**b**) effect of ATT flexible connection’s $\omega_{2}$ on the transfer function, (**c**) effect of LTT flexible connection’s $\xi_{1}$ on the transfer function, (**d**) effect of ATT flexible connection’s $\xi_{2}$ on the transfer function, (**e**) effect of load mass on the transfer function.

The Case3 (LTT and ATT flexible connection) model uses the data in Tables 2 and 4. Figure 12 shows the effect of different intrinsic frequencies ($\omega_{1} (\omega_{p1} )$,$\omega_{t1}$), damping ratios ($\xi_{1} (\xi_{p1} )$,$\xi_{2} (\xi_{t1} )$), and load mass on the transfer function of the Case 3 (LTT and ATT flexible connection) model.

In Fig. 12a, when the intrinsic frequency of the LTT flexible connection is increased from 3 to 9 Hz, the resonance peaks correspond to frequencies all of 0.010 Hz, and the valley amplitudes are all of − 0.11 dB; In Fig. 12b, when the intrinsic frequency of the ATT flexible connection is increased from 3 to 9 Hz, the frequency corresponding to the resonance peak increases from 0.005 to 0.010 Hz, and the valley amplitude decreases from 0.47 to − 0.79 dB; In Fig. 12c, when the damping ratio of the LTT flexible connection is increased from 0.02 to 0.08, the resonance peaks correspond to frequencies of 0.010 Hz and the valley amplitudes are all − 0.11 dB; In Fig. 12d, when the damping ratio of the ATT flexible connection was increased from 0.02 to 0.08, the resonance peaks corresponded to frequencies of 0.010 Hz in both cases, and the valley amplitude increased from − 0.11 to 1.05 dB; In Fig. 12e, when the load mass is increased from 5000 to 15,000 kg, the frequency corresponding to the resonance peak decreases from 0.013 to 0.008 Hz and the valley amplitude decreases from 0.45 to − 0.65 dB; Based on the above data, it can be concluded that in the Case3 (LTT and ATT flexible connection) model, the degree of influence on shaking table performance is not significantly related to the characteristics of the LTT flexible connection, positively related to the intrinsic frequency of the ATT flexible connection, negatively related to the damping ratio of the ATT flexible connection, and positively related to the load mass; The frequency corresponding to the resonance peak is positively correlated with the intrinsic frequency of the ATT flexible connection, negatively correlated with the load mass, and not significantly correlated with the intrinsic frequency of the LTT flexible connection, the damping ratio of the LTT flexible connection, and the damping ratio of the ATT flexible connection; The magnitude of the valley-to-amplitude is positively correlated with the intrinsic frequency of the ATT flexible connection, negatively correlated with the damping ratio of the ATT flexible connection, positively correlated with the load mass, and not significantly correlated with the LTT flexible connection characteristics.

### Analysis of compensation results

**Figure 13:**
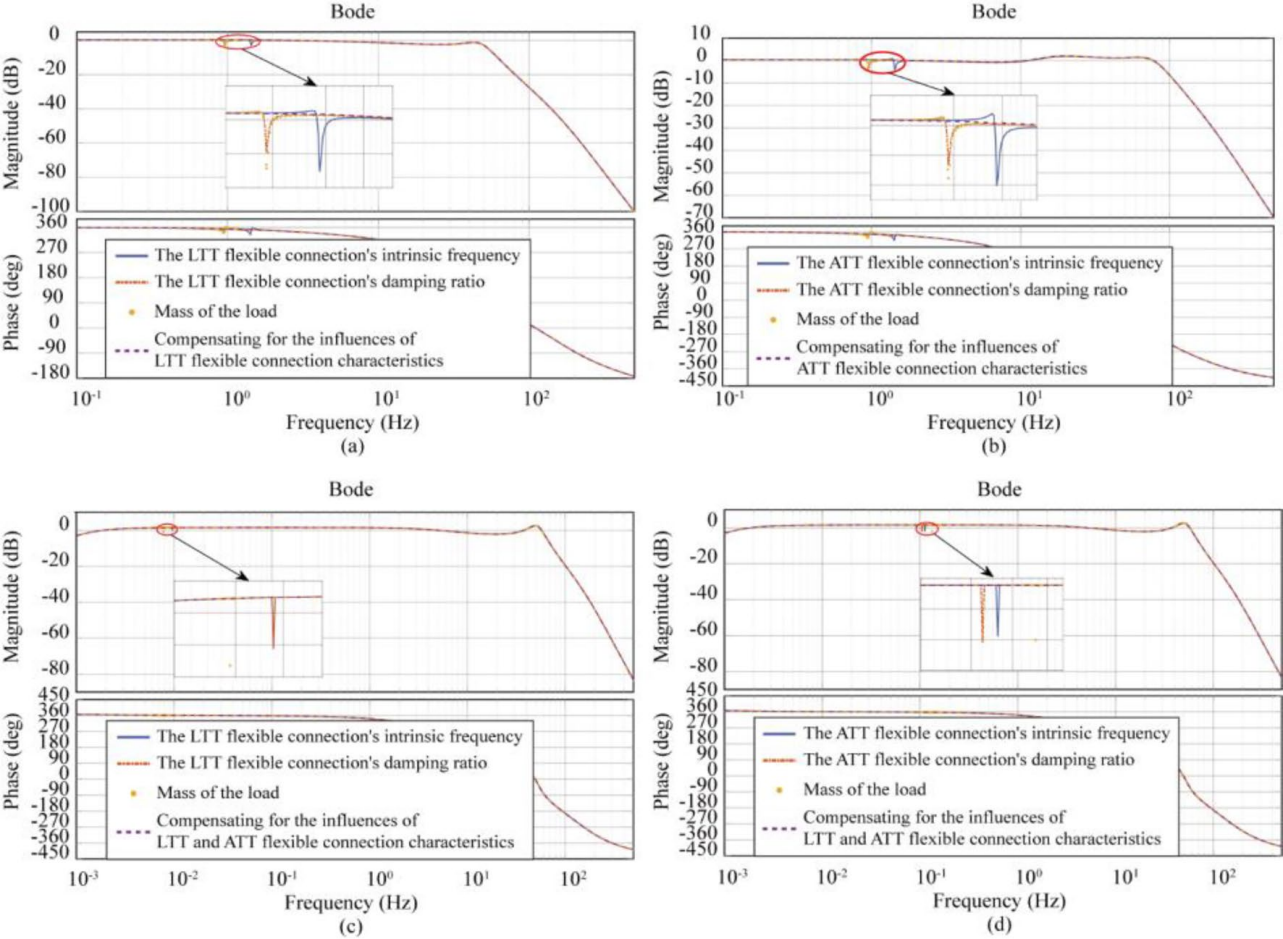
Influence of compensated flexible connection characteristics (**a**) compensated LTT flexible connection characteristics, (**b**) compensated ATT flexible connection characteristics, (**c**, **d**) compensated LTT and ATT flexible connection characteristics.

The above “Effect of different flexible connection characteristics on performance” demonstrates that flexible connections affect the transfer function to varying degrees. The role of flexible connections in Case1, Case2, and Case3 models cannot be ignored. Figure 13 illustrates the frequency response characteristic curve after adding compensation.

In Fig. 13a, the Case1 (LTT flexible connection) model has resonance peaks of magnitude − 3.01 dB at 1.0 Hz and − 3.03 dB at 1.48 Hz, which are compensated to increase in magnitude to 0.32 dB and 0.24 dB, respectively. In Fig. 13b, the Case2 (ATT flexible connection) model has resonance peaks of magnitude − 3.55 dB at 0.95 Hz and − 4.05 dB at 1.43 Hz, which increase in magnitude to 0.26 dB and 0.06 dB, respectively, after compensation; In Fig. 13c and d, Case3 (LTT and ATT flexible connection), which has a negligible effect in the low frequency band (10^–3^ to 10^–2^) and an amplitude of − 3.21 dB at 0.11 Hz and − 3.05 dB at 0.13 Hz, is compensated for by increasing the amplitude to 0.52 dB and 0.49 dB, respectively. From the above data, it can be seen that the significant changes in the frequency response characteristics of the system before and after compensation, the flexible connection characteristics form a resonance peak, while the flexible connection reaction force compensation algorithm eliminates the resonance peak and makes the frequency response curve smoother. The ATT connection flexible connection characteristic has a greater impact on the frequency response characteristics of the system than the LTT flexible connection characteristic. When both flexibility characteristics are considered simultaneously, resonance summits appear within the operating frequency bandwidth when the load mass is reduced to a specific range, while the flexible connection reaction force compensation algorithm remains effective.

**Figure 14:**
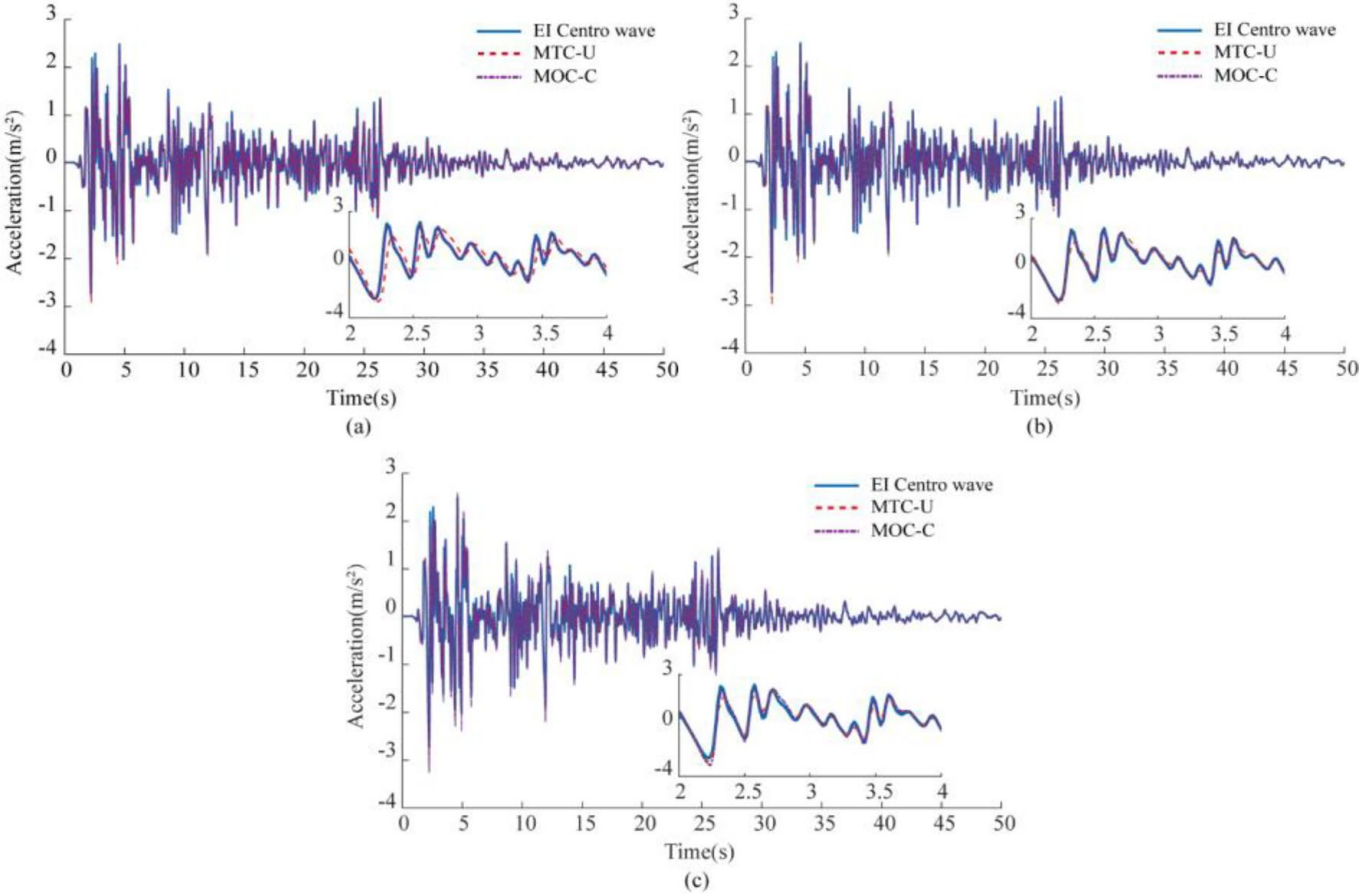
System time domain simulation (**a**) Case1 (LTT flexible connection), (b) Case2 (ATT flexible connection), (**c**) Case3 (LTT and ATT flexible connection).

Figure 13 demonstrates the compensation effect in the frequency domain, which is combined with the previous study to verify whether the compensation algorithm and multiparameter control have a better compensation effect in the time domain. Figure 14 shows the time-domain simulation of the compensated flexible connection characteristics, using the EI Centro wave, Kobe wave, and artificial wave as input signals, and comparing the control effects of the two cases of multiparametric theoretical control without compensation (MTC-U) and multiparametric optimal control with compensation (MOC-C) based on the Case1, Case2 and Case3 models.

Figure 14 demonstrates that the system enhances waveform accuracy by using compensation algorithms and multi-parameter control. The phase delay and amplitude attenuation of the EI Centro wave response are also improved. The errors of the reproduced signals with respect to the input signals are detailed in Table 5, where the maximum and minimum values of the correlation coefficient (CC) are 0.9751 and 0.7108, and the maximum and minimum values of the root-mean-square error (RMSE) are 0.2527 and 0.0821, respectively, for the multicomponent theoretical control (uncompensated) (MTC-U); while for the multicomponent optimal control (compensated) (MOC-C) the maximum and minimum values of correlation coefficient (CC) are 0.9981 and 0.9341, and the maximum and minimum values of root mean square error (RMSE) are 0.0836 and 0.0183, respectively. CC and RMSE were improved and reduced to different degrees, respectively, indicating that the control effect of MOC-C was better than that of MTC-U, confirming the success of the above research strategy.

**Table 5:** Error index.

Control type	Flexible connection type	Error indicator	Acceleration signal type		
			EI-Centro wave	Kobe wave	Artificial wave
MTC-U	LTT	CC	0.8139	0.8502	0.7108
		RMSE	0.2527	0.2192	0.1449
	ATT	CC	0.9662	0.9686	0.9060
		RMSE	0.1090	0.1026	0.0872
	LTT and ATT	CC	0.9737	0.9751	0.9172
		RMSE	0.0966	0.0915	0.0821
MTC-C	LTT	CC	0.9979	0.9981	0.9958
		RMSE	0.0271	0.0248	0.0183
	ATT	CC	0.9971	0.9977	0.9927
		RMSE	0.0321	0.0267	0.0253
	LTT and ATT	CC	0.9874	0.9875	0.9341
		RMSE	0.0836	0.0824	0.0772

## Conclusion and future research

In shaking table systems, interaction forces between the load and the table surface result in poor waveform reproduction accuracy over the frequency bandwidth used. At abode and abroad, many tests have emphasized the importance of specimen characteristics on shaking table performance, and compensation is achieved by designing controllers. However, in the shaking table itself, there are many non-linear factors; and the actuator and table connection, similar to the connection between the load and the table, is flexible. To realize the compensation of the interaction force between the actuator bar, table, and load's flexible connection and improve the system's performance in the time and frequency domains. By simplifying the flexible connection of the actuator, table, and load into a spring-damped system and analyzing the influence of the flexible characteristics on the frequency domain of the system under different conditions, an algorithm for compensating the characteristics of the flexible connection is proposed based on which the resonance peaks existing in the frequency domain are eliminated. The distortion of the waveform is reduced. The compensation algorithm combines multi-parametric control and is stable. However, it has weak compensation in the time domain due to LTT and ATT flexible connections. This may be due to the actuator rod displacement control signal, load influence, or interaction forces canceling each other out. The nonlinear shaking table connection needs further discussion. In summary, the following important conclusions can be obtained by combining the simulation results:

1. Under LTT or ATT flexible connection characteristics, the single-degree-of-freedom load peaks at the first-order frequency; under LTT and ATT flexible connection characteristics, the single-degree-of-freedom load peaks outside the operating bandwidth; based on the multi-parameter optimal control (MOC) can maximally expand the effective frequency bandwidths of the Case1, Case2, and Case3 models to 0.0011–89.10 Hz to improve the system’s Stability.
2. With LTT or ATT flexible connections, the effect on the frequency response characteristics of the system increases with increasing specimen mass and connection intrinsic frequency. As the connection damping ratio increases, its effect decreases; under LTT and ATT flexible connections, the load mass causes changes in the resonance peak frequency and amplitude, the effect of the LTT flexible connection characteristics on the frequency response characteristics of the system is negligible, and the effect pattern of the ATT flexible connection characteristics is similar to that in the case of a single flexible connection.
3. The control strategy combining a flexible connection reaction force compensation algorithm with a multi-parametric optimization algorithm is suitable for shaking table control. In the frequency domain, the use of the compensation algorithm eliminates the resonance peaks caused by the flexible connection, resulting in a smoother frequency response characteristic curve. In the time domain, the MOC-C control strategy results in a CC of up to 0.9981 and an RMSE of down to 0.0183, which improves the waveform reproduction accuracy.

In addition, the model in this paper is built on the basis of the linearized shaking table model, which is an attempt to reduce the connection error of the mechanism, for example, it has better applicability on the single-axis shaking table, and the connection coupling of the multi-axis shaking table is more complicated; and the operation of the mechanism is nonlinear and real-time, which requires high-precision observation to realize the control, for example, the object of observation in this study is the actuator, and for the observation of the table surface and the load that For example, in this study, the observation object is the actuator, and the observation of the table surface and the load is derived from the mechanical equations, and the complexity of the observation object also affects the performance of the shaking table. Future work will focus on these aspects.

### Supplementary Information

Supplementary Information 1.

Supplementary Information 2.

## Data Availability

All data generated or analyzed during this study are included in this published article [and its supplementary information files].
