# Supplementary material for: Influence and compensation of connection characteristics on shaking table control performance
Source: Sci Rep. 2024 Mar 22;14:6860. doi: 10.1038/s41598-024-57239-z (PMC10958025; doi:10.1038/s41598-024-57239-z)
Supplement: Supplementary file 2 — Supplementary Information 2. [file 41598_2024_57239_MOESM2_ESM.docx]

Appendix

We would like to inform you that the research paper's image data is sourced from MATLAB/Simulink, with a specific version of R2022a. The models in the zip file are named after their corresponding image titles. It's essential to note that the models used in Fig. 10 and 11 are identical, and the same applies to Fig. 12 and 13. To provide an example of the diagramming process, please see below.

Step 1: Click "Linearization Manager" under "APP" in the MATLAB ribbon to set the input and output points of the recognition system, as shown in Figure 1.


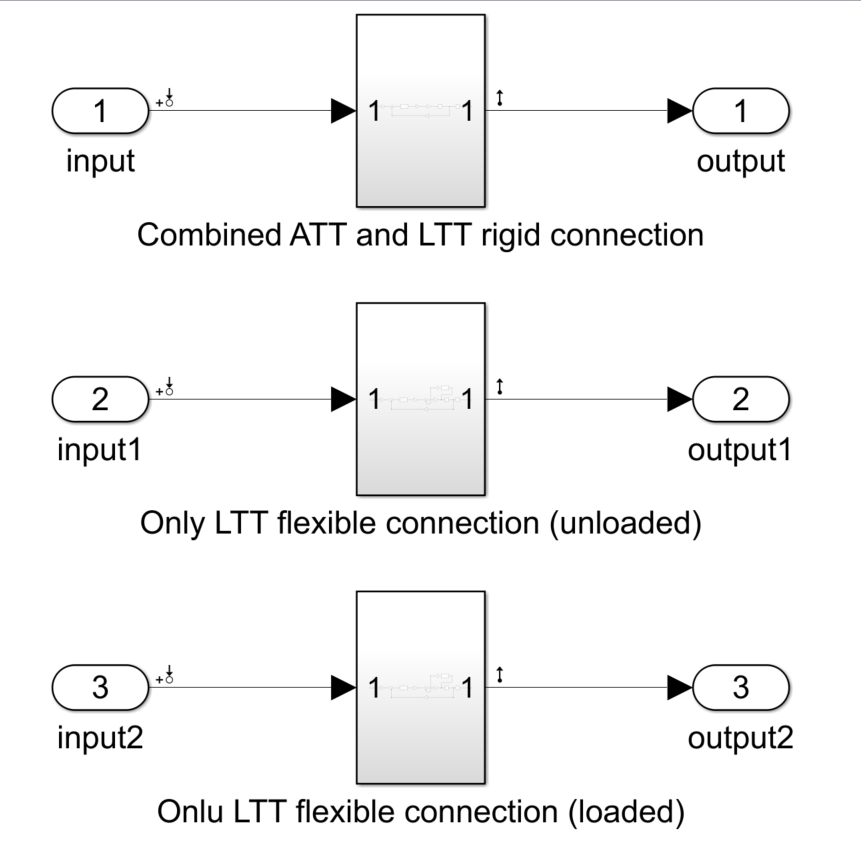


Figure 1 Diagram of Step 1

**Step 2:** In the "LINEARIZATION" function area, click "Model Linearizer", select Bode from the map.


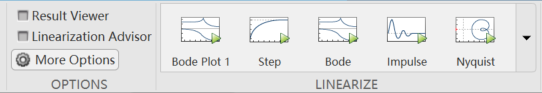


Figure 2 Diagram of Step 2

After the above two steps, the frequency response characteristic curve of the corresponding model can be obtained.

Regarding the zero-pole diagram of the system, take Fig 11 as an example.

**Step 1:** Click "Linearization Manager" under "APP" in the MATLAB ribbon to set the input and output points of the recognition system, as shown in Figure 1.

**Step 2:** In the "LINEARIZATION" function area, click "Model Linearizer", select the Pole-Zero Map out of the map.


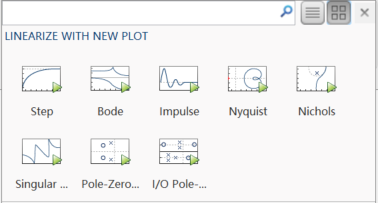


Figure 3 Diagram of Step 2

Regarding the time domain diagram of the system, Fig. 32 is used as an example.

**Step 1:** Input the data in the zip file named "CO2_RSN6_IMPVALL.I_I-ELC180" in the form of a numerical matrix, and then import the data into the "From Workspace" module. Then import the data into the "From Workspace" module and set the sampling time.

**Step 2:** Set the running time, click "Run", click "scope" module to view the time domain map.
